# Characterization of Flaxseed Oil Bimodal Emulsions Prepared with Flaxseed Oil Cake Extract Applied as a Natural Emulsifying Agent

**DOI:** 10.3390/polym12102207

**Published:** 2020-09-26

**Authors:** Emilia Drozłowska, Artur Bartkowiak, Łukasz Łopusiewicz

**Affiliations:** Center of Bioimmobilisation and Innovative Packaging Materials, Faculty of Food Sciences and Fisheries, West Pomeranian University of Technology Szczecin, Janickiego 35, 71-270 Szczecin, Poland; artur-bartkowiak@zut.edu.pl (A.B.); lukasz.lopusiewicz@zut.edu.pl (Ł.Ł.)

**Keywords:** flaxseed, emulsions, protein, flaxseed gum, emulsifying properties, agricultural residues

## Abstract

Currently, a majority of oilseeds plants are converted into byproducts and waste materials during processing. Press cakes are rich in valuable biopolymers, such as proteins and polysaccharides (fiber, lignans, etc.). In this study flaxseed oil cake extract (FOCE) was used to stabilize flaxseed oil-in-water emulsions. The effect of FOCE with various flaxseed oil concentrations (10–50% *v*/*v*) on several physicochemical properties of emulsions, such as stability, rheology, color and particle size was investigated. The rheological parameters suggested that all samples were non-Newtonian fluids, whereas particle size measurements and calculation SPAN index provided information about the broadness of emulsions particle size distribution. FOCE was able to efficiently stabilize oil/water interfaces with a high oil content. Results obtained for FOCE were compared with effects for synthetic emulsifier (Tween 80) and separated FOCE compounds (flaxseed gum and flaxseed protein). FOCE emulsifying activity is a result of different water-holding and oil-binding capacities of flaxseed gum and protein. This result is an intriguing conclusion regarding the necessity for using pure emulsifiers, showing the possibility of using a bio-based extract containing biopolymers, which is part of the principles of circular economy and the idea of zero-waste. The results give the opportunity to use FOCE as an ingredient in efficient flaxseed oil emulsions stabilizer for food applications.

## 1. Introduction

An emulsion is a thermodynamically unstable complex system that generally needs be stabilized with various emulsifiers [1]. Many traditional food products such as milk, butter, dressings (especially mayonnaise), yogurt or ice cream are all emulsions [2,3,4]. Recent studies have been focused on green methods of developing emulsions based on compounds derived from plant biopolymers such as polysaccharides (cellulose, lignin or starch) and proteins (soy, pea, rapeseed, flaxseed, etc.) [5]. Increasing demand for healthy functional food additives has forced the food industry to focus on searching for products of this nature. The food industries are forced to explore different raw materials or to use new technologies/food processes to develop new functional products, because ingredients from animal sources are extensively exploited, although they are considered less environmental friendly compared to the ingredients derived from plants [6,7]. The food market represents a large part of the global economy and is growing every year. Hand-in-hand, this economic sector is now also responsible for approx. 1.3 billion tons of waste per annum. This waste, from fruit, vegetable, and food, includes waste generated during all aspects of food production: cleaning, processing, cooking, and packaging. However, some of these waste products and/or by-products can be important sources of bioactive compounds, such as phenolic compounds, vitamins, carotenoids, pigments, oils, and various biopolymers such as polysaccharides (including dietary fiber) and proteins. Valorization of food industry waste and plant residues represents an attractive path towards obtaining biodegradable materials and achieving “zero waste” goals. Crop residues such as bran, husk, bagasse, and fruit seeds are utilised as a potential raw material in bioprocesses as they provide the food industry with substrates such as emulsifying agents, antioxidant compounds and media growth for microorganisms, which supply them with essential nutrients [8,9]. One of the industries generating large amounts of by-products is the oil industry. For instance, press cakes (derived from rapeseed, sunflower, flaxseed or soy), are by-products that constitute a valuable source of ingredients like proteins, polysaccharides (including fiber) and bioactive compounds such as flavonoid and polyphenolic compounds [10,11]. There are two types of oil cakes: edible and non-edible. Non-edible oil cakes such as castor cake, karanja cake, neem cake are often used as organic nitrogenous fertilizers or to protect the plants from soil nematodes, insects, and parasites; thereby offering great resistance to infection [12]. Edible cakes have a high nutritional value, i.e., their protein content ranges from 15% to 50%. Currently, oil cakes are mainly used to feed animals such as poultry, fish and swine. The composition of cake extracts strongly depends on their extraction method and plant growth conditions. Oil cakes have potential benefits when used as substrates in developing bioprocesses for the production of organic chemicals and biomolecules [12]. However, the utilization of some oil cakes as human food/food additives is limited due to the natural presence of antinutritional compounds, for example flaxseed cake contains cyanogenic glycosides compounds (such as linustatin, neolinustatin, linamarin, lotaustralin). However, they can be significantly reduced by various processing methods such as thermal, microbial or enzymatic treatment [8,13,14,15]. Flaxseed oil cake (FOC) is an example of residual by-product from the oil industry. It is produced every year in large quantities (417,000t in 2016, EU countries) [16]. FOC is a valuable and cheap by-product, rich in omega-3 fatty acids, proteins, soluble and insoluble fibers, phytoestrogenic lignans, different types of antioxidant compounds, vitamins (A, C, D and E), and minerals (Mg, K, Na, Fe, Cu, Mn and Zn). However, is commonly poorly utilized or used as livestock feed [17]. The valorization of FOC is a relatively new issue, however there are reports about its application in food technology [8,9,18,19], polymer technology [20], that indicate the potential of this valuable agro-industrial by-product in the idea of circular economy and sustainable development. Flaxseed oil (oil produced from flaxseed *Linum usitatissimum* L.) is a source of valuable fatty acids like α-linolenic acid (ALA), which consumption may prevent cardiovascular disease, insulin resistance and non-alcoholic fatty liver disease [21]. However, flaxseed oil a is very susceptible to lipid oxidation in the presence of environmental factors such as oxygen, heat and light, which adversely affect its bioactivity [17,21,22]. It is known that some oils are used in their emulsified form by applying proteins and/or polysaccharides as emulsifying agents, which increases their stability and the possibility of including them in the diet [23,24]. Natural emulsifying agents based on polymeric compounds such as polysaccharides or proteins have many advantages when compared with synthetic polymers. They are biodegradable, non-toxic and easily available in the environment [8]. Plant-based proteins have a significantly lower global warming potential when compared with proteins obtained from animal sources. The current trend for natural and more suitable bio-based emulsifying agents is a good area for enhancing the development of obtaining valuable components from the oil industry by-products under the influence of market trends connected to zero waste and circular economy. In the previous studies, it was reported that FOC can be used in the production of low-fat mayonnaises and, after the spray drying process, it is possible to use it to obtain powders with antioxidant and emulsifying properties [2,15]. The present work strongly focuses on the emulsifying properties of a FOC-based water extract and describe the type of emulsions containing flaxseed oil. Bimodal emulsions are unique systems that are non-common phenomenon, little known and poorly described in the literature. Whereas monomodal emulsions are characterized by one particle size distribution, bimodal emulsions are characterized by having two different and controlled droplet size and distributions [25,26]. The bimodal character of particle size distribution was described for animal protein (whey protein concentrate) with rapeseed oil [27]. There is a lack of reports about this type of emulsions in food science and the available works only indicate that this phenomenon is quite common for oilseed proteins [22,28,29,30]. Bimodal emulsions due to their mechanical and chemical influences have unique rheological properties for example high flow adaptability and relatively low bulk viscosity for semi-solid products. Additionally, bimodal distribution tends to reduce the viscosity of a particulate suspension. The rheological behaviour of this type of emulsions is important for transporting viscous products in food processing without risk of pipes system plugging and can be used to prepare emulsion blends containing two or more different polymers [31]. Bimodal emulsions have many applications in various areas e.g., in oil transportation, petrochemical, food, and cosmetic industries. Emulsion environmental or formulation parameters change continuously during manufacturing and storage. The bimodal system suspensions represent a number of food products that often have high calorie densities, such as sauces, desserts and dressings, especially in semi-solid food products with reduced caloric value.

To the best of our knowledge, there have been no reports on the utilization of flaxseed oil cake obtained via cold-press technique to produce a protein and polysaccharide rich extract and its application as an emulsifying agent for bimodal flaxseed oil emulsions. Thus, the aim of the presented study was to use the aqueous extract from flaxseed oil cake, to obtain emulsions with 10–50% *v/v* content of flaxseed oil as well as to examine their stability and describe in this case these unique emulsions. Emulsions reported in this study are novel in the food technology sector and the evaluation of FOCE in practical use as an emulsifier has not been previously reported in a scientific way. Additionally, information obtained from this research may provide useful insights into the rational formulation of semi-solid food systems (with pasta or cream consistency) stabilized by flaxseed protein-gum mixtures.

## 2. Materials and Methods

### 2.1. Materials

Flaxseed oil cake (FOC) was kindly donated by ACS Sp. z o.o. (Bydgoszcz, Poland) and cold-pressed flaxseed oil was obtained via a cold press technique (Olejarnia Niwki, Zwoleń, Poland). According to the manufacturer’s information, the proximate composition of FOC was as follows: 80.5% solids content, 42% protein content, 28% carbohydrates content, 6.3% fiber content, 6.1% fat content, 4.5% ash content. Sodium dodecyl sulphate (SDS), ethanol (96%), hydrochloric acid, Tween 80, Sudan III, sodium hydroxide, Benedict’s reagent, phenol solution (5%), sulfuric acid (96%), glucose, bovine serum albumin were purchased from Sigma Aldrich (Darmstadt, Germany). All reagents were of analytical grade.

### 2.2. Preparation of Flaxseed Oil Cake Extract (FOCE), Flaxseed Protein (FP) and Flaxseed Gum (FG) Extraction

The procedure to obtain Flaxseed Oil Cake Extract (FOCE) consists of several steps [15]. Initially, FOC was ground and mixed with distilled water in a 1:10 ratio (*v/v*). Then, the mixture was heated at 90 °C for 1 h with constant stirring (250 rpm). The next step was cooling down the mixture to room temperature and centrifugation (2500 rpm) for 30 min at 20 °C (MPW-352, MED Instruments, Warsaw, Poland). The supernatant (FOCE) was filtered under vacuum and homogenized for 5 min with a homogenizer (SilentCrusherM, Heidolph, Schwabach, Germany) at 12,000 rpm. Crude flaxseed gum (FG) was extracted with 96% ethanol in ratio 1:1 according to Wang et al. [32] with a slight modification. The extracted flaxseed gum solution was filtered through 40-mesh filter paper, then dried at 55 °C for 12 h. The flaxseed protein (FP) solution was obtained by the precipitation in the isoelectric point. The pH of FOCE was adjusted to 4.2 with 0.1 M HCl and centrifuged at 3000 rpm for 10 min [33]. The resulting precipitate of FP was washed with a ten-fold volume of distilled water and again centrifuged at 3000 rpm for 10 min. Finally, the FP pellet was dried at 55 °C for 12 h.

### 2.3. FOCE Characterization

The total solids content was determined by drying the FOCE at 105 °C for 24 h [34]. The protein content of FOCE was determined by the micro-Biuret method with bovine serum albumin as a reference sample [35]. The carbohydrate content was evaluated based on the phenol-sulfuric acid reaction using glucose as a reference sample [36]. All measurements were performed with the use of a Thermo Scientific Evolution 220 spectrophotometer (Thermo Fisher Scientific Inc., Waltham, MA, USA). In order to confirm the presence of particular chemical moieties in FOCE, FTIR analysis was performed by attenuated total reflection with an FTIR spectrometer operating in ATR mode (Model 100 spectrophotometer, Perkin Elmer, Waltham, MA, USA) [15]. In brief, FOCE was dried for 24 h at room temperature, pulverized in a mortar, then 100 mg of sample was placed directly on the ray-exposing stage of the ATR accessory, and scanned at a range between 650 cm^−1^ and 4000 cm^−1^ (64 scans and 1 cm^−1^ resolution). The obtained spectrum was baseline corrected and analyzed using the Spectrum^TM^ software (v.10, Perkin Elmer).

### 2.4. Determination of Water-Holding and Oil-Binding Capacities of FOCE, FG and FP

Water holding capacity (WHC) and oil binding capacity (OBC) of FOCE, FG and FP were determined according to Gong et al. with a slight modification [15,37]. To determine WHC, 1 g of a particular sample (W_0_) was placed in Falcon tubes and weighed together with the tubes (W_1_). Then, 10 mL of distilled water was added to the samples and was vigorously vortexed for 10 s. After thorough wetting, the samples were allowed to stand at room temperature for 30 min and then centrifuged at 3000 rpm for 20 min. The supernatants were decanted and the tubes containing the sediments were weighed (W_2_). The WHC was calculated according to the formula:

$$\mathrm{WHC}=\frac{W_{2}-W_{1}}{W_{0}}$$

To determine OBC, 1 g of sample (W_0_) was placed in a Falcon tubes and weighed together with the tubes (W_1_). Then, 10 mL of rapeseed oil was added to the samples and they were vigorously vortexed for 10 s. The next steps were identical as in WHC determination. The OBC was calculated according to the formula:

$$\mathrm{OBC}=\frac{W_{2}-W_{1}}{W_{0}}$$

### 2.5. Emulsion Preparation and Emulsifying Properties Measurements 

Emulsions with 10–50% content (volume disperse fraction in emulsions) of the FO phase were prepared in two steps. In the first step, FOCE was mixed with flaxseed oil (10–50% *v*/*v*) for 5 min with a magnetic stirrer (IKA, Staufen, Germany). The next step included homogenization for 5 min with a homogenizer (SilentCrusherM, Heidolph) at 2000 rpm. Similarly, the solutions of FG (6.5 mg/mL) and FP (14 mg/mL) were prepared in distilled water, based on the previously measured contents of each component in FOCE. The emulsions with FG and FP solutions were prepared in the same way as with FOCE. The emulsifying properties of FOCE, FG and FP were evaluated in freshly prepared emulsions as described elsewhere [38]. Twenty µL of each emulsion were mixed with 5 mL of 0.1% SDS solution and vortexed. Then, the absorbance was measured at 500 nm. Emulsions with 3% content of Tween 80 and without any emulsifying agent (consisting only of water and FO) with the 10–50% of the FO (volume disperse in FO/W emulsions), were used as a control sample. The stability of emulsions was calculated as the corresponding Emulsion Stability Index (ESI) according to the formula [38,39]:

$$\mathrm{ESI}=\frac{A_{0}}{A_{0}-A_{10}}\times t$$

where: *A*_0_ is the initial absorbance (0 min), *A*_10_ is the absorbance after 10 min and *t* is the time between measurements (10 min).

The Emulsion Activity Index (EAI) was calculated according to the following formula:

$$\mathrm{EAI}=\frac{2\times 2.303\times A_{0\;}\times DF}{C\times 1\;\mathrm{cm}\times \mathrm{oil}\;\mathrm{fraction}\;\mathrm{volume}\times 10000}$$

where: *A*_0_ is the initial absorbance (0 min), *DF* is the dilution factor (200), and *C* is the dry matter of FOCE, FG, FP or Tween 80 in the emulsion (g/mL).

### 2.6. Determination of Emulsion with FOCE, FP, FG and Tween 80 Particle Size Distribution and SPAN Index

Particle size distribution measurements of emulsions with FOCE, FP, FG and Tween 80 were performed using a Mastersizer 2000 (Malvern Instrument Ltd., Worcestershire, UK) [15]. Emulsions were diluted with 0.1% SDS solution, gently stirred and dispersed in distilled water (stirred speed—2000 rpm) until an obscuration rate of 10% was obtained. The optical properties of the sample were defined as follows: refractive index 1.500 and absorption 1.00. Droplet size measurements were reported as the volume-weighted mean diameter d_4,3_ = ∑n_i_d^4^_i_/∑n_i_d_i_^3^ where n_i_ is the number of droplets of diameter d_i_. The dispersion index (SPAN) was calculated according to the formula:

$$\mathrm{SPAN}=\frac{d_{90}-d_{50}}{d_{10}}$$

where: d_10_, d_50_ and d_90_ are the diameters at 10%, 50% and 90% of cumulative volume, respectively.

### 2.7. Optical Microscope Observations of Emulsions with FOCE, FP, FG and Tween 80

Samples of emulsions with FOCE, FP, FG and Tween 80 were mixed in ratio 1:1 with 0.1% SDS, Sudan III was added as an oil dye, and the mixtures were coated on the glassy flat. The samples were observed with a microscope (OptaTech, Warsaw, Poland) at a magnification of 10× at room temperature. The pictures of emulsions structures were obtained by a digital camera connected to the microscope [2].

### 2.8. Color Determination of Model Emulsions

The color of the emulsions was measured by a Konica Minolta CR—5 colorimeter with the Hunter LAB color system (Konica Minolta, Osaka, Japan). Color coordinates were expressed as lightness (L), redness/greenness (a), and yellowness/blueness (b). 

### 2.9. Rheological Measurements and Interfacial Tension

The flow experiments were performed using a rheometer (AR G2, TA Instruments Ltd., New Castle, DE, USA). The temperature was kept at 20 °C during the measurements. The viscosity of the emulsions was measured with a stainless-steel cone plate geometry of 62 mm diameter and 1° cone angle. The steady-state flow procedure was carried out in the range of 0.1 to 100 s^−1^. The data of the rheological measurements were recorded by TA Rheology Advantage Data Analysis equipment software V 5.4.7. Experimental flow curves were fitted to the Herschel–Bulkley (H–B) model:

$$\tau =\tau_{0}+k{\dot{\gamma }}^{n}$$

where: *τ*—the shear stress (Pa), *τ*_0_—the yield stress (Pa), *γ̇*—the shear rate (s^−1^), *k*—the consistency index (Pa·s^n^), *n*—the flow index [2,40]. 

The interfacial tension between FOCE, FP, FG, and FO was determined by the use of the Du Noüy ring method using a tensiometer STA-1 (Sinteface, Berlin, Germany). The results were compared with the interfacial tension between water and flaxseed oil, 3% Tween 80 solution and flaxseed oil. Interfacial tension was calculated according to the formula:

$$\gamma =\frac{Fmax}{4\Pi R\beta }$$

where: *γ* is the interfacial tension, R is the radius of the ring (18.85 mm), and β (13.71) is a correction factor depending on the dimension of the ring and the density of the liquid involved.

### 2.10. Statistical Analysis

All the experiments were replicated three times. The results are expressed as a mean ± standard deviation. All the data were subjected to a one-way analysis of variance (ANOVA) test using the software Statistica 13.0 (StatSoft, Kraków, Poland). Significant differences between the means were determined by Fisher’s NIR multiple comparison tests at *p* < 0.05.

## 3. Results and Discussion

### 3.1. The Proximate Composition of FOCE

**Figure 1:**
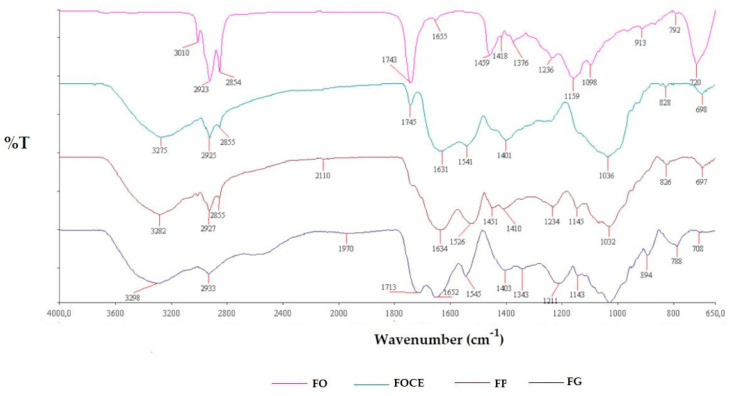
The FTIR spectra of flaxseed oil (FO), flaxseed oil cake extract (FOCE), flaxseed protein (FP), and flaxseed gum (FG).

It was noticed that FOCE had 3 ± 0.2% of dry matter, and contained 14 ± 0.2 mg/mL of proteins, 6.5 ± 0.2 mg/mL of carbohydrates and 9.5 ± 0.02 mg/mL of other extractable compounds. FOCE was a homogenous liquid containing proteins and polysaccharides, which fractions adsorb onto the oil-water surface during the emulsification process [37,40]. FTIR spectroscopy was used in order to confirm the presence of particular chemical moieties in FOCE (Figure 1). The characteristic CH_3_ and CH_2_ stretching peaks at 2925 cm^−1^ and 2855 cm^−1^, respectively, were observed [15,37,41]. At 1631 cm^−1^, a strong amide I band was noticed which is attributed to C=O stretching vibrations, N–H stretching vibrations, N–H bending vibrations, and C–N bending vibrations in proteins [15,37,41]. The same peaks were noticed in sample FP. Peak recorded for FG at 1652 cm^−1^ is assigned to C=O of glucuronic acid [42]. FG consists of two major polysaccharides fractions: a neutral arabinoxylan, which is substantially free of uronic acid, and acidic pectic-like polysaccharide. A band at 3275 cm^−1^ was assigned to N–H stretching vibrations of the primary amide structure of proteins, as well as O–H stretches, C–H stretches, and residual water [15,37,41]. Peaks recorded at 1036 cm^−1^ (angular deformation of =CH and =CH_2_ bonds), 828 cm^−1^ (deformation of C-H and CH_2_), and 698 cm^−1^ (structural situation of the pyranose ring) can be attributed to FG polysaccharides [15,37,41]. The peak at 1745 cm^−1^ was observed both in FOCE and FO, which is characteristic to different carbonyl groups such as aldehydes, ketones and carboxylic acids, and can be attributed to small content of oil residues in FOCE. FOC contains up to 40% protein composed of amino acids such as glutamic acid, arginine, valine, leucine, tyrosine, and phenylalanine [12,13,14]. Flaxseed proteins (FP) are divided into two main fractions: salt-soluble 11–12S globulins and water-soluble 1.6–2S albumins, which are referred to as linin and conlinin. According to Kaushik et al. the isoelectric point (pI) of flaxseed protein isolate is 4.2 [33]. FP amino acid composition and nutritional value is comparable to soy protein and exhibits favorable oil and water absorption, emulsifying activity and stability [2,15,43]. Mucilage plays a barrier role during the flaxseed oil cake extraction (FOCE) process, but it is not necessary to reject this component because, from the standpoint of specific functional requirements, the preparation of the protein-rich extract with mucilage may be more desirable than its rejection by expensive processes, since both protein and mucilage can be utilized in mixtures [5,22,23,24,27,28]. Geerts et al. [44] reported that in the case of emulsion stabilization, multicomponent fractions from yellow pea were capable to stabilize O/W emulsions. In similar work with a sunflower cake, Karefyllakis et al. indicated that separation of pure proteins is not necessary to use them as emulsifiers and that potentially coarse protein mixtures have good emulsifying properties [28]. Pham et al. described strong emulsifying properties of flaxseed complexes consisting of protein, polysaccharides and phenolic compounds [45]. It is widely known, that flaxseed mucilage could also be used in food applications [46,47]. For example, mixes of flaxseed protein concentrate and mucilage (flaxseed protein concentrate containing mucilage or FPCCM), which are extracted from a defatted cake, have been already used in the food sector in China in the meat and ice-cream industries [22]. The mucilage obtained from flaxseed contains L-galactose, D-xylose, L-arabinose, L-rhamnose, and D-galacturonic acid and it exhibits a high water holding capacity and oil binding ability, which are significant during the emulsion formulation process [45,47,48]. Polysaccharides included in mucilage are responsible for the formulation of a multiform structure, and better resistance to environmental stresses. From a nutritional standpoint, flaxseed mucilage is a good source of dietary fiber and has many health benefits such as the preventing of diabetes, obesity, or colon cancer, decreasing blood cholesterol levels and improving insulin sensitivity [8,49]. Flaxseed polysaccharides are called flaxseed gum (FG), which is composed of 80% neutral and acidic polysaccharides. The neutral part of polysaccharides are composed of xylose, glucose, arabinose, and galactose, while acidic monosaccharides consist of rhamnose, galactose, fucose, and galacturonic acid [47,50,51,52]. Moreover, protein and mucilage from FOCE present various physico-chemical and structural properties such as emulsification, foaming and oil binding, which imparts food products with a satisfying appearance, taste, texture and rheological behaviour [53]. The aim of adding proteins as emulsifiers is to facilitate of the formation of smaller oil droplets during homogenization. In this case, the interfacial tension at the oil-water interfacial is lowering. The addition of proteins also increases stability during the increasing repulsive colloidal interaction.

### 3.2. Water-Holding and Oil-Binding Capacities of FOCE, FP, FG, and Interfacial Tension

**Figure 2:**
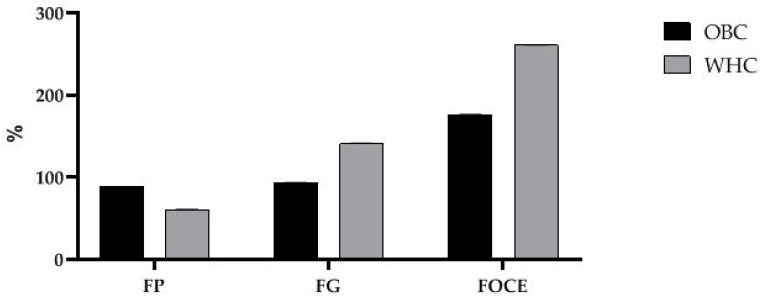
Water Holding Capacity and Oil Binding Capacity of FG, FP and FOCE. FG—flaxseed gum solution; FP—flaxseed protein solution, FOCE—flaxseed oil cake extract.

Water-holding capacity (WHC) and oil-binding capacity (OBC) have a significant influence on selected compounds/mixtures potential as emulsifying agents. The WHC and OBC values of FOCE, FG and FP are presented in Figure 2. It could be observed that FP exhibited significantly higher OBC than FG, whereas FG has stronger ability to water holding. Indeed, high WHC of FG was reported by Sun et al., and is linked with highly branched polysaccharides structure and their chemical composition (many functional acyl groups) [47]. This contributes to FG gel water binding via electrostatic interactions and the hydrogen bonds would be possible. WHC of FG reported by Wang et al. is comparable with WHC reported in present work (140.69 ± 0.76%) [53]. FOCE is a complex of FG and FP and showed strong ability for binding water (260.70 ± 0.28%) as well as oil (175.97 ± 0.37%). Indeed, high WHC and OBC of spray-dried FOCE powders were reported [15]. Obtained results are in a good agreement with the findings of other works about protein-polysaccharide systems [54,55]. 

One of the most important steps in the emulsification process is the absorption of molecules at the oil-water interfacial area [5]. To effectively reduce the interfacial tension, proteins should have the ability to orient their hydrophobic residues to the oil phase and hydrophilic residues to the aqueous phase [56]. Table 1 summarizes the results of the interfacial tension measurements. It was noticed that FOCE/FO interfacial tension (26.89 ± 0.64 mN/m] was significantly lower (*p* < 0.05) than those for FG and FP (35.89 ± 0.54 mN/m and 38.71 ± 0.09 mN/m, respectively). Similar surface tension reduction by FG was described by Ding et al. [57]. The effect of FOCE on interfacial tension is caused by higher interfacial adsorption [56]. Similar results were reported for potato protein-sunflower oil emulsions (25 mN/m) [58]. FOCE consists of FG, FP and other water-soluble compounds which may affect its strong interfacial tension reduction properties. The ability to stabilize emulsions due to the reduction of the interfacial tension was also shown in the work of Kuhn et al. [59]. A comparable observation was made for sunflower press cake extract, which exhibited strong interfacial activity, decreasing the interfacial tension from an initial 27 mN/m to an equilibrium value of 8.5 mN/m [23].

### 3.3. Emulsion Stability

**Figure 3:**
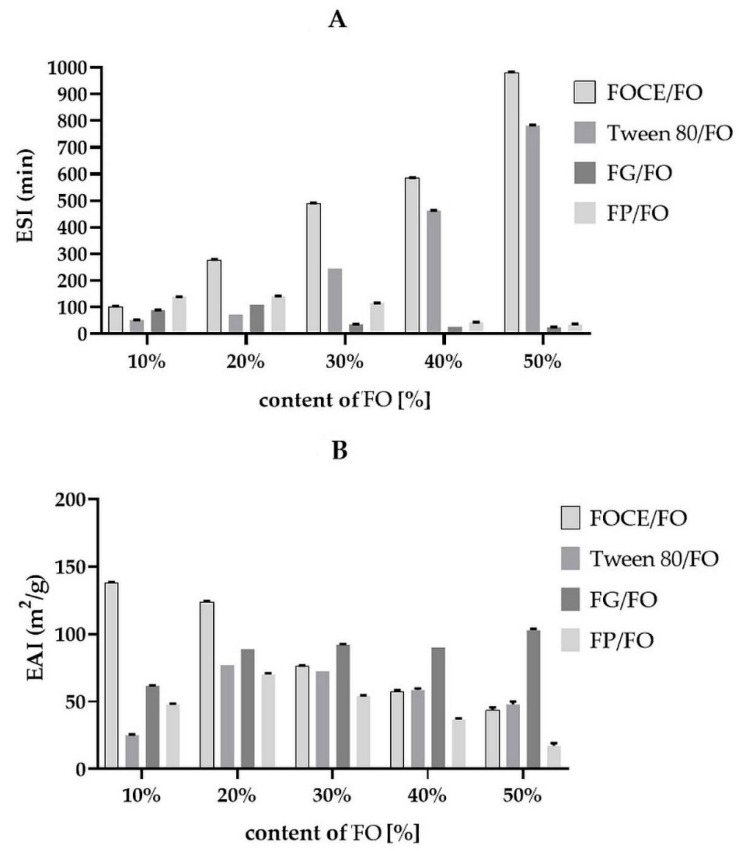
(**A**)—Emulsion Stability Index (ESI) of emulsions with FOCE, 3% Tween 80, FP and FG solution prepared with various content of FO; (**B**)—Emulsion Activity Index (EAI) of emulsions with FOCE, 3% Tween 80, FP and FG solution prepared with various content of FO. FOCE- flaxseed oil cake extract; FG- flaxseed gum solution; FP- flaxseed protein solution; Vertical bars represent ± SD.

Freshly prepared emulsions with FOCE, FP and FG were apparently homogenous and stable against phase separation, therefore their stability was expressed by two indicators: ESI and EAI. Emulsions without any emulsifier (consisted only of water and FO) were unstable and they were not suitable for further research. The ESI and EAI parameters are strongly influenced by several factors such pH at which emulsions are made, protein purity, extraction method, and other technological processes, which determine the solubility of protein fractions as well as their oil binding capacity [15]. As shown in Figure 3, when the flaxseed oil fraction volume increased in FOCE-emulsions, the ESI increased significantly (*p* < 0.05). The highest ESI (981 ± 1.41 min) was noticed for the emulsion containing 50% of FO. The lowest EAI (138 ± 1.03 m^2^/g) was observed for emulsion containing 50% of FO, which is higher than the one reported for soy protein (56–99 m^2^/g), a common natural emulsifying agent [47]. However, this result is lower than the EAI reported by Kaushik et al. [33] for coacervates with pure flaxseed protein and flaxseed gum (375 m^2^/g), as well as for flaxseed protein isolate (220 m^2^/g), reported by Krause et al. [60].

The ability of FOCE to stabilize emulsions with 10–50% content of flax oil was compared to model emulsions with 3% of Tween 80, which is dedicated to food emulsions with high content of oil, and emulsions without any emulsifying agent (only water and oil). It could be observed, that FOCE showed a tendency to obtaining higher ESI index and higher or similar level of EAI index like emulsions with Tween 80. All emulsions without emulsifying agent were unstable with average ESI value 11.83 min. In this case the EAI could not be evaluated. The emulsion activity index of flaxseed extracts is influenced not only by the oil content but also strongly depends on the extraction method. One of the steps of the extraction procedure involved heating, which can affect the emulsifying properties of proteins [61]. In fact, in previous studies it was reported that denaturated FOCE had higher emulsifying properties in mayonnaise systems than native the one [2]. Similarly, it was found that along with the increase of FOCE spray drying temperature, the emulsifying properties of the obtained powders increased [15]. Figure 3 presents the ESI and EAI indexes of emulsions prepared with the two main components of FOCE: FG and FP. It could be observed a clear tendency of FP to long term stabilization with low EAI, whereas FG showed the ability to high EAI for a short time. In both cases the emulsion stability index was decreasing with increasing oil content. It could be concluded, that in emulsions with FOCE polysaccharides are responsible for high emulsification capability and proteins are a factor providing long term stability. Those observations are in line with phenomenon described by Nasrabad et al. [5]. The authors suggested that emulsions only with FP have strong ability to flocculation and emulsions with mixed systems like FOCE showed higher stability.

### 3.4. Droplet Size and SPAN Index

**Figure 4:**
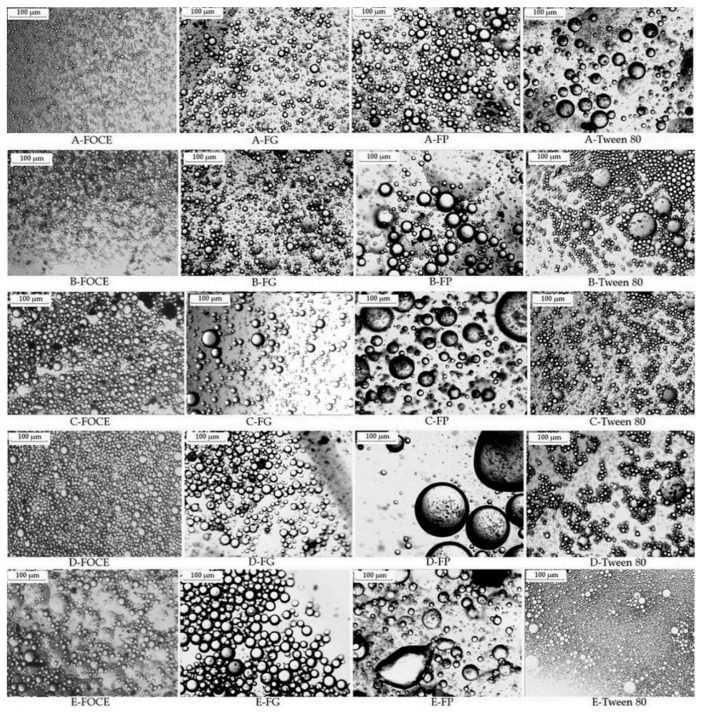
Optical microscopic images of emulsions prepared with various FO and FOCE contents, FP, FG and Tween 80. FOCE—flaxseed oil cake extract; FG—flaxseed gum solution; FP—flaxseed protein solution; (**A**–**E**) emulsions prepared with 10%, 20%, 30%, 40% and 50% oil phase, respectively.

**Figure 5:**
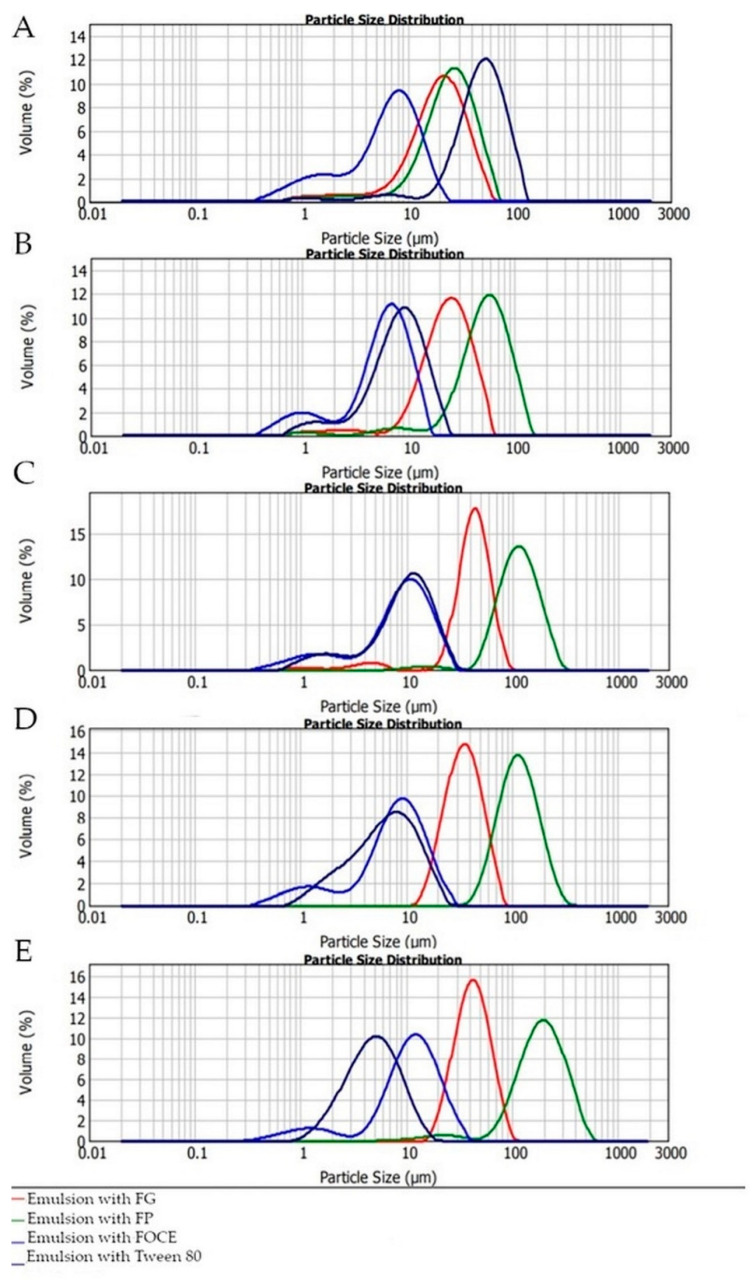
Particle size distribution of emulsions prepared with various content of FO with FOCE, FG, FP and Tween 80. FOCE—flaxseed oil cake extract; FG—flaxseed gum solution; FP—flaxseed protein solution; (**A**–**E**): emulsions prepared with 10%, 20%, 30%, 40% and 50% oil phase, respectively. Values are means ± standard deviation of triplicate determinations.

Droplet size always affects the stability of the emulsion, because emulsions with controlled particle size exhibit better stability [53]. The volume of FO fraction significantly influenced the emulsions particle size and SPAN index as well (*p* < 0.05). The highest D_4,3_ in FOCE—emulsions was observed for sample E, containing 50% of FO (12.19 ± 0.30 µm). It could be observed that droplet size obtained with FOCE is higher than for emulsions with 3% content of Tween 80. The D_4;3_ parameters for FG and FP separately are higher in each case than for FOCE. As shown in Table 2, the smallest particle size and SPAN index were noticed for emulsion B, containing 20% of FO (6.21 ± 0.10 µm and 1.646 ± 0.10 µm, respectively). Those results are in line with the captures from the microscope presented in Figure 4. Similarly to the results of Sun et al. [47] the deposition of the concentrated emulsion, which could be observed in the optical microscope, does not influence the stability of the emulsions, which is demonstrated by the results for the ESI index. It can be a result of the thickening effect of the polysaccharides included in FOCE, which played an essential role to ensure stability [53]. In emulsion E with FOCE an Ostwald ripening could be observed. This phenomenon is often observed in water-in-oil emulsions where oil molecules diffuse through the aqueous phase and form larger oil droplets [62]. The main reasons for that are insufficient emulsifier concentration required to cover the oil droplets and increased coalescence due to increased oil concentration. Another reason of Ostwald ripening is largely dictated by the solubility of the oil in the continuous phase. The aqueous phase solubility of an oil decreases linearly with oil molar volume [63]. Increasing the volume of oil in emulsions enhances the tendency to Ostwald ripening and 50% content of FO in probably the limitation area. Based on the results presented in Figure 4 and Table 2 it can be observed that the droplet size increase is corresponding to increasing oil volume in emulsions. In fact, the increase of droplet size of O/W emulsions with the increase in oil phase concentration is reported [64]. The dispersion index of emulsion (SPAN) prepared with FOCE ranged from 1.646 to 1.865. Statistically significant differences were observed for emulsions with 10%, 20%, and 30% of FO (*p* < 0.05). Lowering the SPAN index is in line with the results of the EAI and ESI assessments and shows that increasing the content of oil could provide a seemingly stable emulsion with a low emulsion activity index and very low homogeneity with relatively big particles. As presented in Figure 5, all emulsions exhibited a bimodal character, which so far has been described for O/W emulsions stabilized with flaxseed protein [53,65] and soy protein [66]. Bimodal emulsions are characterized by having two different and controlled droplet sizes and distribution [26]. In this study, the bimodal character of emulsions is an effect of two different main components of FOCE (protein and polysaccharides fractions). According to Querol et al. the bimodal emulsions have much higher stability than monomodal ones [25]. The authors suggested that an optimal formulation to obtain a stable emulsion should contain a first small-sized fraction of about 1 µm and the second fraction of about 5 µm with a proportion of 1:2, respectively. In Figure 5 values d(0,1) and d(0,5) are presented, which agrees with the cited thesis and could be an explanation for the high stability of emulsions. A similar bimodal structure was observed for emulsions with sunflower cake extract mixed with oil by Karefyllakis et al., who described a similar mechanism of the particle distribution [28]. The droplet size distribution started from the same range of 0.3 μm, and the second major peak appeared at about 5 μm [28]. It could be concluded that bimodal distribution is an effect of synergistic action of all FOCE components. 

In fact, as can be seen in Figure 5, emulsions prepared with FP or FG showed monomodal character. Similar tendency was described for emulsions prepared with pea protein [67]. Bimodal emulsions have very specific particle behavior mechanisms. According to Sourdet et al. [68], a higher size class of particles (in the second peak of bimodal distribution) could be due to an earlier coalescence of fat globules. Other results of bimodal distribution could be the formation of covalently bound aggregates between proteins adsorbed onto fat droplets [22].

### 3.5. Emulsions Rheological Characteristics and Interfacial Tension

Table 3 summarizes the rheological character of emulsions based on the Herschel-Bulkey model. According to Sun and Gunasekaran, increasing the oil-phase volume has a direct influence on K value, because increasing oil phase content enhances droplet interactions and emulsion stability [69]. Yield stress level is a moment of breaking the stress curve when the emulsion starts to flow and the magnitude of this parameter informs about the potential stability of the emulsion [2]. The lowest yield stress in FOCE-emulsions (0.0014 ± 0.003 Pa) was observed for emulsion A with 10% of oil, whereas the highest yield stress (0.6590 ± 0.001 Pa) was noticed for sample D (content 40% of the oil phase). Lower yield stress and viscosity for sample E showed the point where the emulsifying properties diminish. There might not have been enough protein and polysaccharide particles present to cover all the oil droplets during the homogenization. The flow behavior of all samples, besides FG and FP solutions, emulsion A with FP and emulsions D and E with Tween 80, showed a pseudoplastic and non-Newtonian character. According to Sun and Gunasekaran, the volume of the oil phase has no significant influence on flow behavior index value (n) [69]. Similar effects for rheological parameters were described by Wang et al. for flaxseed protein [53]. Flaxseed gums exhibited the highest yield stress, which indicated they high stability, but showed very low viscosity. It could be observed a tendency that the highest values of viscosity and consistency index are observed for emulsions with FOCE, lower for emulsions with FG and the lowest for samples with FP. Similar phenomenon is noted for yield stress, only exception is observed in case sample E with the highest content of FO, indicating the moment when the emulsion particles size is too high to obtain a stable system. This is corresponding to microscopic observations (where an Ostwald ripening was observed) and effects of D_4;3_ measurements. Emulsions with 3% *w/w* Tween 80 showed lower yield stress than samples with FOCE and its components. It indicated that, FP, FG and FOCE as a natural emulsifier could be used to emulsification systems with high content of oil. 

### 3.6. Emulsion Color

The results of the color determination could be useful for evaluating emulsion stability because these structures are very sensitive to color changes which are connected to aggregation [15]. The overall appearance of an emulsion is determined by a combination of light scattering and absorption phenomena, which are responsible for the turbidity or lightness of an emulsion and color, respectively [52,70]. As can be seen in Table 4, lightness of FOCE and FG emulsions decreased significantly with increasing oil content (*p* < 0.05), which is concomitant with the results of the particle size measurements. The aggregation of the fat droplets within the emulsions due to the presence of high content of the oil phase may, therefore account for the observed decrease in lightness [70,71]. However, this was not observed for FP emulsions, and is probably linked with high particles size when compared with emulsions prepared with FOCE and FG. 

## 4. Conclusions

This article explored the possibility of using a bio-based FOCE containing biopolymers as a natural emulsifier, and characteristics of FOCE/FO emulsions in comparison to emulsions prepared with two main FOCE fractions (flaxseed gum and protein), was well as with Tween 80. The information obtained in this study revealed that the FOCE can be efficiently used for FO emulsification, forming bimodal emulsions, and showing good ability to reduce interfacial tension. The emulsifying properties of FOCE are a result of its complex nature, and different water-holding and oil-binding capacities of the two main fractions: flaxseed protein and flaxseed gum. It was demonstrated that flaxseed protein exhibited higher oil-binding capacity than flaxseed gum, whereas flaxseed gum had stronger ability to water holding. Oil-phase volume fraction significantly affected the droplet size, and rheological properties of the emulsions, but in all cases the bimodal character was a constant feature of the samples. The utilization of valuable agro-industrial by-product is in line of the principles of circular economy and the idea of zero-waste.

## Figures and Tables

**Table 1:** The interfacial tension between various phases (FOCE, water, FG, FP, 3% Tween 80) and flaxseed oil.

Sample *	γ [mN/m]
FOCE/FO	26.89 ± 0.64 ^a^
W/FO	47.63 ± 0.03 ^b^
FG/FO	35.89 ± 0.54 ^c^
FP/FO	38.71 ± 0.09 ^d^
3% Tween 80/FO	19.89 ± 0.26 ^e^

* FOCE/FO—interfacial tension between FOCE and flaxseed oil; W/FO—interfacial tension between water and flaxseed oil; FG/FO—interfacial tension between flaxseed gum solution and flaxseed oil; FP/FO—interfacial tension between flaxseed protein solution and flaxseed oil; 3% Tween 80/FO—interfacial tension between 3% Tween 80 solution in water and flaxseed oil. Values are means ± standard deviation of triplicate determinations. Means with different letters in the same column are significantly different at *p* < 0.05.

**Table 2:** D_4,3_ and SPAN values of emulsions prepared with various content of oil.

**D_4.3_ (µm)**				
**Sample**	**FOCE**	**FG**	**FP**	**Tween 80**
**A**	7.13 ± 0.00 ^d^	21.58 ± 0.02 ^e^	26.73 ± 0.10 ^e^	52.76 ± 0.11 ^a^
**B**	6.21 ± 0.10 ^e^	25.06 ± 0.02 ^d^	57.36 ± 0.97 ^d^	8.94 ± 0.02 ^b^
**C**	8.72 ± 0.03 ^c^	37.30 ± 0.13 ^c^	122.17 ± 0.05 ^c^	5.96 ± 0.01 ^c^
**D**	9.72 ± 0.01 ^b^	43.36 ± 0.10 ^b^	125.04 ± 0.72 ^b^	4.51 ± 0.05 ^d^
**E**	12.19 ± 0.30 ^a^	44.99 ± 0.42 ^a^	205.98 ± 0.30 ^a^	4.03 ± 0.10 ^e^
**SPAN (-)**				
**A**	1.864 ± 0.00 ^a^	1.503 ± 0.02 ^a^	1.451 ± 0.03 ^d^	1.332 ± 0.10 ^d^
**B**	1.646 ± 0.10 ^c^	1.208 ± 0.02 ^b^	1.362 ± 0.00 ^b^	1.195 ± 0.05 ^e^
**C**	1.803 ± 0.01 ^b^	1.069 ± 0.10 ^c^	1.154 ± 0.05 ^a^	1.672 ± 0.03 ^b^
**D**	1.865 ± 0.03 ^a^	0.988 ± 0.15 ^d^	1.154 ± 0.02 ^a^	1.892 ± 0.06 ^a^
**E**	1.770 ± 0.30 ^b^	0.809 ± 0.02 ^e^	1.354 ± 0.03 ^c^	1.569 ± 0.00 ^c^

FOCE—flaxseed oil cake extract; FG—flaxseed gum solution; FP—flaxseed protein solution; A–E emulsions prepared with 10%, 20%, 30%, 40 and 50% oil phase, respectively Values are means ± standard deviation of triplicate determinations. Means with different lowercase in the same column are significantly different at *p* < 0.05.

**Table 3:** Rheological parameters of samples based on Herschel–Bulkley model.

**τ_y_ [Pa]**				
	**FOCE**	**FG**	**FP**	**Tween 80**
	0.0010 ± 0.001 ^Af^	0.3456 ± 0.001 ^Bb^	0.0045 ± 0.001 ^Cf^	-
**A **	0.0014 ± 0.003 ^Ae^	0.0005 ± 0.001 ^Bf^	0.1436 ± 0.004 ^Cc^	0.0007 ± 0.001 ^Bc^
**B**	0.0041 ± 0.001 ^Ad^	0.0007 ± 0.000 ^Be^	0.0172 ± 0.005 ^Ce^	0.0001 ± 0.012 ^De^
**C**	0.1450 ± 0.000 ^Ac^	0.0203 ± 0.003 ^Bd^	0.0958 ± 0.003 ^Cd^	0.0020 ± 0.006 ^Dd^
**D**	0.6590 ± 0.001 ^Aa^	0.3209 ± 0.002 ^Bc^	0.1471 ± 0.006 ^Cb^	0.0810 ± 0.001 ^Da^
**E**	0.3570 ± 0.002 ^Ab^	0.6987 ± 0.003 ^Ba^	0.6987 ± 0.003 ^Ba^	0.0048 ± 0.002 ^Cb^
**Viscosity [Pa·s]**				
	**FOCE**	**FG**	**FP**	**Tween 80**
	0.0540 ± 0.005 ^Af^	0.0007 ± 0.000 ^Bf^	0.0006 ± 0.000 ^Cf^	-
**A **	0.0960 ± 0.000 ^Ae^	0.0060 ± 0.004 ^Be^	0.0005 ± 0.003 ^Ce^	0.0021 ± 0.001 ^Dd^
**B**	0.1590 ± 0.001 ^Ad^	0.0095 ± 0.003 ^Bd^	0.0029 ± 0.001 ^Cd^	0.0036 ± 0.000 ^Dc^
**C**	1.1640 ± 0.003 ^Ac^	0.0272 ± 0.001 ^Bc^	0.0057 ± 0.000 ^Cc^	0.0035 ± 0.001 ^Dc^
**D**	3.9080 ± 0.001 ^Aa^	0.0594 ± 0.000 ^Bb^	0.0234 ± 0.000 ^Ca^	0.0040 ± 0.005 ^Db^
**E**	3.6320 ± 0.001 ^Ab^	0.1336 ± 0.002 ^Ba^	0.0081 ± 0.005 ^Cb^	0.0065 ± 0.000 ^Da^
**K [Pa·s^n^]**				
	**FOCE**	**FG**	**FP**	**Tween 80**
	0.076 ± 0.001 ^Af^	0.0001 ± 0.000 ^Bf^	0.0003 ± 0.001 ^Ce^	-
**A **	0.146 ± 0.004 ^Ae^	0.0071 ± 0.002 ^Be^	0.0001± 0.003 ^Cf^	0.0021 ± 0.003 ^Dd^
**B**	0.211 ± 0.001 ^Ad^	0.0125 ± 0.001 ^Bd^	0.0042 ± 0.000 ^Cd^	0.0053 ± 0.002 ^Db^
**C**	1.083 ± 0.000 ^Ac^	0.0471 ± 0.000 ^Bc^	0.0076 ± 0.008 ^Cc^	0.0044 ± 0.000 ^Dc^
**D**	1.799 ± 0.001 ^Aa^	0.0987 ± 0.006 ^Bb^	0.0562 ± 0.000 ^Ca^	0.0011 ± 0.001 ^Dd^
**E**	1.420 ± 0.001 ^Ab^	0.2330 ± 0.000 ^Ba^	0.0116 ± 0.000 ^Cb^	0.0065 ± 0.003 ^Da^
**n [-]**				
	**FOCE**	**FG**	**FP**	**Tween 80**
	0.88 ± 0.001 ^Aa^	1.00 ± 0.001 ^Ba^	1.13 ± 0.001 ^Cb^	-
**A **	0.82 ± 0.002 ^Ac^	0.97 ± 0.003 ^Bb^	1.24 ± 0.001 ^Ca^	0.99 ± 0.001 ^Dc^
**B**	0.85 ± 0.001 ^Ab^	0.94 ± 0.001 ^Bc^	0.93 ± 0.000 ^Cd^	0.93 ± 0.001 ^Ce^
**C**	0.53 ± 0.000 ^Ad^	0.85 ± 0.000 ^Bd^	0.94 ± 0.004 ^Cc^	0.96 ± 0.003 ^Dd^
**D**	0.43 ± 0.004 ^Ae^	0.82 ± 0.000 ^Be^	0.77 ± 0.000 ^Ce^	1.24 ± 0.001 ^Da^
**E**	0.86 ± 0.000 ^Ab^	0.72 ± 0.005 ^Bf^	0.93 ± 0.000 ^Cd^	1.00 ± 0.000 ^Db^

FOCE—flaxseed oil cake extract; FG—flaxseed gum solution; FP—flaxseed protein solution; A–E: emulsions prepared with 10%, 20%, 30%, 40% and 50% oil phase. Values are means ± standard deviation of triplicate determinations. Means with different lowercase in the same column are significantly different at *p* < 0.05. Means with different uppercase in the same raw are significantly different at *p* < 0.05.

**Table 4:** Color values of emulsions prepared with various content of flaxseed oil.

	**L**			
	**FOCE**	**FG**	**FP**	**Tween 80**
	53.34 ± 0.01 ^Af^	32.60 ± 0.01 ^Bf^	31.59 ± 0.02 ^Bf^	-
**A **	89.53 ± 0.03 ^Aa^	89.90 ± 0.00 ^Ba^	79.19 ± 0.51 ^Bc^	81.95 ± 0.23 ^Ce^
**B**	89.43 ± 0.01 ^Ab^	89.42 ± 0.00 ^Ab^	78.36 ± 0.69 ^Cb^	90.89 ± 0.01 ^Cc^
**C**	87.83 ± 0.02 ^Ac^	81.46 ± 0.01 ^Bc^	79.24 ± 0.29 ^Cd^	91.24 ± 0.01 ^Ca^
**D**	87.52 ± 0.03 ^Ad^	80.59 ± 0.01 ^Bd^	80.29 ± 0.22 ^Cc^	91.18 ± 0.02 ^Cb^
**E**	83.28 ± 0.02 ^Ae^	80.08 ± 0.10 ^Be^	79.19 ± 0.51 ^Ce^	90.18 ± 0.01 ^Cd^
	**a**			
	**FOCE**	**FG**	**FP**	**Tween 80**
	2.12 ± 0.00 ^Aa^	−1.67 ± 0.02 ^Bf^	−1.55 ± 0.01 ^Cf^	-
**A **	0.50 ± 0.00 ^Ac^	−1.00 ± 0.00 ^Be^	−0.90 ± 0.01 ^Ce^	−0.29 ± 0.04 ^De^
**B**	0.27 ± 0.01 ^Af^	0.41 ± 0.00 ^Bd^	0.33 ± 0.32 ^Cd^	0.49 ± 0.01 ^Dc^
**C**	0.53 ± 0.00 ^Ab^	2.63 ± 0.03 ^Bb^	2.56 ± 0.11 ^Cc^	0.74 ± 0.01 ^Da^
**D**	0.34 ± 0.01 ^Ae^	2.27 ± 0.01 ^Bc^	2.81 ± 0.04 ^Cb^	0.52 ± 0.01 ^Db^
**E**	0.47 ± 0.00 ^Ad^	3.97 ± 0.01 ^Ba^	3.65 ± 0.03 ^Ca^	0.07 ± 0.01 ^Dd^
	**b**			
	**FOCE**	**FG**	**FP**	**Tween 80**
	36.01 ± 0.01 ^Aa^	−6.44 ± 0.02 ^Bf^	−6.57 ± 0.01 ^Cf^	-
**A **	24.18 ± 0.06 ^Ae^	32.20 ± 0.17 ^Be^	32.54 ± 1.03 ^Ce^	40.24 ± 1.48 ^Da^
**B**	21.94 ± 0.04 ^Af^	41.56 ± 0.21 ^Bd^	42.07 ± 2.50 ^Cd^	30.74 ± 0.04 ^Db^
**C**	26.23 ± 0.01 ^Ac^	52.42 ± 0.42 ^Bc^	55.46 ± 1.46 ^Cc^	30.24 ± 0.19 ^Dc^
**D**	25.20 ± 0.03 ^Ad^	55.18 ± 0.11 ^Bb^	58.82 ± 0.51 ^Cb^	29.49 ± 0.02 ^Dd^
**E**	26.70 ± 0.01 ^Ab^	61.50 ± 0.06 ^Ba^	61.66 ± 0.30 ^Ca^	28.53 ± 0.03 ^De^

FOCE—flaxseed oil cake extract FG—flaxseed gum solution; FP—flaxseed protein solution; A–E: emulsions prepared with 10%, 20%, 30%, 40% and 50% oil phase, respectively. Values are means ± standard deviation of triplicate determinations. Means with different lowercase in the same column are significantly different at *p* < 0.05. Means with different uppercase in the same raw are significantly different at *p* < 0.05.
